# A Dual Zinc plus Arginine formulation attenuates the pathogenic properties of *Porphyromonas gingivalis* and protects gingival keratinocyte barrier function in an *in vitro* model

**DOI:** 10.1080/20002297.2020.1798044

**Published:** 2020-08-04

**Authors:** Amel Ben Lagha, Ying Yang, Harsh M. Trivedi, James G. Masters, Daniel Grenier

**Affiliations:** a Oral Ecology Research Group, Faculty of Dentistry, Université Laval, Quebec City, QC, Canada; b Colgate-Palmolive Technology Center, Piscataway, NJ, USA

**Keywords:** Arginine, dentifrice, epithelial barrier, gingipain, periodontal disease, periodontitis, *Porphyromonas gingivalis*, toothpaste, zinc

## Abstract

**Background and objectives:**

*Porphyromonas gingivalis*, a late colonizer of the periodontal biofilm, has been strongly associated with the chronic form of periodontitis. The aim of this study was to investigate the effects of a Dual Zinc plus Arginine formulation (aqueous solution and dentifrice) on the pathogenic properties of *P. gingivalis* and the barrier function of an *in vitro* gingival epithelium model.

**Results:**

The Dual Zinc plus Arginine aqueous solution and dentifrice inhibited the hemolytic and proteolytic activities of *P. gingivalis*. The Dual Zinc plus Arginine aqueous solution and dentifrice enhanced the barrier function of an *in vitro* gingival epithelium model as determined by a time-dependent increase in transepithelial electrical resistance and decrease in paracellular permeability. This was associated with an increased immunolabeling of two important tight junction proteins: zonula occludens-1 and occludin. The deleterious effects of *P. gingivalis* on keratinocyte barrier function as well as the ability of the bacterium to translocate through a gingival epithelium model were attenuated in the presence of either Dual Zinc plus Arginine aqueous solution or dentifrice.

**Conclusions:**

The Dual Zinc plus Arginine formulation may offer benefits for patients affected by periodontal disease through its ability to attenuate the pathogenic properties of *P. gingivalis* and promote keratinocyte barrier function.

## Introduction

The oral cavity is colonized by a wide range of microbial species, mainly bacteria, which interact with each other and with host cells, contributing to physiological and pathological conditions. Dental biofilm is initially formed by Gram-positive facultative anaerobic cocci and rods, including *Streptococcus* and *Actinomyces* species. As the dental biofilm matures, colonization shifts toward strictly anaerobic Gram-negative bacterial species that contribute to the subgingival biofilm that initiates periodontal disease (gingivitis, periodontitis) [1]. The progression and severity of periodontal disease is modulated by a limited group of bacteria that challenge mucosal and immune cells, leading to the establishment of a chronic inflammatory condition [2]. More specifically, periodontitis is characterized by irreversible and progressive destruction of the supporting tissues surrounding the teeth, including the alveolar bone.

Although periodontal disease is considered a multifactorial polymicrobial infection, *Porphyromonas gingivalis* is suspected to be one of the most important causative agents of the chronic form of this disease [3,4]. This keystone bacterial species has been suggested to induce the transition from a symbiotic microbial community to a dysbiotic microbiota [5]. *P. gingivalis* contributes to the pathogenesis of periodontitis through the expression of a wide range of virulence factors, including cysteine proteinases, also known as gingipains, that disturb host defense mechanisms, degrade tissue proteins, and modulate the host inflammatory response [6,7].

The oral epithelium creates a physical protective barrier between the underlying connective tissue and invasive periodontal pathogens and their toxic products in the oral environment, and thus plays an active role in the maintenance of periodontal health [8,9]. The intercellular tight junctions, which are composed of specialized transmembrane proteins that regulate transepithelial permeability, are the primary cellular determinant of epithelial barrier integrity and function [10]. *P. gingivalis* has developed different strategies to compromise the structural and functional integrity of the oral epithelium. Using specific gingipain inhibitors and gingipain-deficient mutants of *P. gingivalis*, Groeger et al. [11] provided evidence that these proteolytic enzymes are involved in the degradation of cell-to-cell junctions and the disruption of the epithelial barrier. Once the integrity of the oral epithelium is disrupted, *P. gingivalis*, along with other periodontal pathogens, can reach deeper connective tissues and trigger a marked pro-inflammatory response that modulates tissue destruction. Bacteria and their toxins can also enter the bloodstream, migrate to extra-oral sites, and cause systemic complications [12,13]. Taken the above into consideration, conditions or substances with the ability to attenuate the *P. gingivalis*-mediated destructive process or to promote the oral epithelial barrier integrity may be of high interest for maintaining or recovering oral health.

In previous human clinical studies [14,15], a dentifrice formulation containing zinc (zinc oxide, zinc citrate) and arginine, known as Dual Zinc plus Arginine, was reported to significantly decrease oral bacterial counts as well as plaque and gingivitis parameters compared to a regular fluoride dentifrice. The aim of the present study was to investigate the effects of the Dual Zinc plus Arginine formulation as an aqueous solution and in a fluoride dentifrice on the pathogenic properties of *P. gingivalis* and on the barrier function of an *in vitro* gingival epithelium model.

## Materials and methods

### Dual Zinc plus Arginine formulation

Zinc oxide and zinc citrate trihydrate were obtained from U.S. Zinc (Houston, TX, USA) and Jost Chemical (St. Louis, MO, USA), respectively. L-arginine (free form) was purchased from Ajinomoto (Tokyo, Japan). A mixture containing 0.96% zinc (zinc oxide, zinc citrate) and 1.5% arginine was freshly prepared in sterile distilled water and is referred to as the Dual Zinc plus Arginine aqueous solution. Unless indicated otherwise, the Dual Zinc plus Arginine aqueous solution was used at dilutions of 1/500, 1/1000, and 1/2000 (v/v). A dentifrice containing 0.96% zinc (zinc oxide, zinc citrate), 1.5% arginine, and 1450 ppm fluoride as sodium fluoride in a silica base marketed by Colgate-Palmolive Co. (Toronto, Canada) as Colgate Total toothpaste formula was also used. A zinc- and arginine-free control fluoride dentifrice was also tested. Unless indicated otherwise, the Dual Zinc plus Arginine dentifrice and control fluoride dentifrice were used at dilutions of 1/500, 1/1000, and 1/2000 (w/v). At the dilutions used, the amounts of zinc and arginine in the Dual Zinc plus Arginine aqueous solution and the Dual Zinc plus Arginine dentifrice were comparable. The pH of the aqueous solution was 10, while that of the dentifrice was 8.2; however, at the dilutions used, the buffer capacity of the culture tissue medium and the assay solutions brings the final pH at around 7.2. When the Dual Zinc plus Arginine aqueous solution and dentifrice were inoculated onto Todd-Hewitt agar plates (THA; Becton, Dickinson and Company, Sparks, MD, USA), no microbial contamination was observed (data not shown).

### Bacteria and growth conditions

*P. gingivalis* ATCC 33277 was grown in an anaerobic chamber (80% N_2_, 10% CO_2_, 10% H_2_) for 24 h at 37°C in Todd-Hewitt broth (Becton, Dickinson and Company) supplemented with 0.001% (w/v) hemin and 0.0001% (w/v) vitamin K (THB-HK).

### Hemolytic assay

Fresh sheep red blood cells (Nutri-Bact, Terrebonne, QC, Canada) were harvested from heparinized whole blood by centrifugation (600 x g for 5 min), washed three times in phosphate-buffered saline (PBS; pH 7.0), and suspended in PBS to a concentration of 2% (v/v). Equal volumes (1 ml) of red blood cells, *P. gingivalis* cells (optical density at 660 nm [OD_660_] = 1.0 in PBS), and two-fold serial dilutions of the Dual Zinc plus Arginine (aqueous solution and dentifrice) or the control dentifrice were mixed together. PBS replaced the bacteria in the negative control. Following an incubation at 37°C for 4 h, the mixtures were incubated at 4°C for 1 h and were then centrifuged (10 000 x g for 5 min) prior to recording the absorbance of the supernatants at 540 nm (A_540_). Assays were performed in triplicate in two independent experiments and the means ± standard deviations (SD) were calculated.

### Proteolytic assay

To determine the effects of the Dual Zinc plus Arginine aqueous solution and dentifrice on the proteinase activity of *P. gingivalis*, a 48-h culture was centrifuged at 10 000 x *g* for 10 min and the supernatant was collected. Assay mixtures containing equal volumes of *P. gingivalis* culture supernatant, the fluorescent substrate collagen DQ^TM^ (100 µg/ml; Molecular Probes, Eugene, OR, USA), and the Dual Zinc plus Arginine aqueous solution or dentifrice were prepared and incubated for 2 h at 37°C. The fluorescence corresponding to collagen degradation was monitored at time 0, 30, 60, 90, and 120 min using a Synergy 2 microplate reader (BioTek Instruments, Winooski, VT, USA), with the excitation and emission wavelengths set at 495 nm and 525 nm, respectively. Test compounds or the fluorescent substrate alone were used as controls. Leupeptin (1 µM) was used as a positive inhibitory control. Assays were performed in triplicate in two independent experiments and the means ± SD were calculated.

### Human gingival keratinocyte culture

The previously characterized B11 immortalized human gingival keratinocyte cell line [16] was used to investigate the effects of the Dual Zinc plus Arginine aqueous solution and dentifrice on keratinocyte barrier integrity. Keratinocytes were cultivated in keratinocyte serum-free medium (K-SFM; Life Technologies Inc., Burlington, ON, Canada) supplemented with growth factors (50 µg/ml of bovine pituitary extract and 5 ng/ml of human epidermal growth factor) and 100 µg/ml of penicillin G-streptomycin at 37°C in a 5% CO_2_ atmosphere.

### Transepithelial electrical resistance assay

The tight junction integrity of the B11 gingival keratinocyte barrier was assessed by determining the transepithelial electrical resistance (TER) using the procedure described by Gumbiner and Simons [17]. Briefly, B11 keratinocytes were seeded onto Costar™ Transwell™ clear polyester membrane inserts (6.5-mm diameter; 0.4-µm pore size; Corning Co., Cambridge, MA, USA) at 3 × 10^5^ cells per insert. The basolateral and apical compartments were filled with 0.6 ml and 0.1 ml of complete K-SFM, respectively, and the cultures were incubated for 3 days at 37°C in a 5% CO_2_ atmosphere. The conditioned medium was then replaced with fresh antibiotic-free K-SFM. Following a further incubation (16 h), the TER values were measured using an Ohm/voltmeter (EVOM2; World Precision Instruments, Sarasota, FL, USA) at 0, 2, 6, 24, and 48 h. Resistance values were calculated in Ohms (Ω)/cm^2^ by multiplying the resistance values by the filter surface area. Results were expressed as a percentage of the basal control values measured at time 0 h (100% values) for each condition. To investigate the effects of the Dual Zinc plus Arginine aqueous solution and dentifrice on tight junction integrity, the medium in the apical compartment was supplemented with the test compounds. The effect of these treatments on cell viability was assessed using an MTT (3-[4,5-diethylthiazol-2-yl]-2,5diphenyltetrazolim bromide) colorimetric assay, according to the manufacturer’s instructions (Roche Diagnostics, Laval, QC, Canada).

The effect of *P. gingivalis* on the tight junction integrity of the gingival keratinocyte barrier model was evaluated by monitoring TER at 0, 6, 24, and 48 h. *P. gingivalis* cells in antibiotic-free K-SFM were added to the apical compartment at a multiplicity of infection (MOI) of 10^4^. The protective effects of adding the Dual Zinc plus Arginine aqueous solution or dentifrice were evaluated by adding them to the apical compartment at the same time as the *P. gingivalis* cells. All the above assays were performed in triplicate in three independent experiments and the means ± SD of a representative set of data is presented.

### Paracellular permeability assay

The ability of the test compounds to enhance or protect gingival keratinocyte barrier integrity was further assessed by monitoring the paracellular transport of fluorescein isothiocyanate (FITC)-conjugated 4.4-kDa dextran (FD-4; Sigma-Aldrich Canada Co., Oakville, ON, Canada) across the keratinocyte layer. Briefly, B11 cells were cultured on Transwell™ filters, and FD-4 (1 mg/ml in culture medium) was added to the apical compartment in the presence of the test compounds. The presence of FD-4 in the basolateral compartment was determined at 0, 2, 6, 24, and 48 h by measuring the fluorescence (relative fluorescence units [RFU]; excitation wavelength 495 nm; emission wavelength 525 nm) using a Synergy 2 microplate reader. The effects of *P. gingivalis* (MOI = 10^4^) on paracellular permeability and the protective effects of the Dual Zinc plus Arginine aqueous solution and dentifrice were assessed under the conditions described above. Assays were performed in triplicate in three independent experiments and the means ± SD of a representative set of data is presented.

### Immunofluorescent staining of zonula occludens-1 and occludin

Gingival keratinocytes treated for 48 h as described above (test compounds ± *P. gingivalis*) were immunostained for two tight junction proteins (zonula occludens [zo-1] and occludin) using a previously described protocol [18]. The localization of the tight junction proteins in B11 cells was visualized using an Olympus FSX100 fluorescence microscope and FSX-BSW imaging software (Olympus, Tokyo, Japan). This experiment was performed three times; a representative experiment and a representative field of this experiment is presented.

### P. gingivalis *translocation assay*

B11 gingival keratinocytes cultured as described above were seeded at 2.25 × 10^5^ cells per insert in high-throughput screening (HTS) 96-well Costar™ Transwell™ plates (8-µm pore size; Corning Co.), which were placed in Costar™ black receiver plates (Corning Co.). The basolateral and apical compartments were filled with 0.235 ml and 0.075 ml of K-SFM, respectively. Following a 48-h incubation, the conditioned medium was replaced with antibiotic-free K-SFM. To determine the ability of *P. gingivalis* to penetrate the keratinocyte layer, FITC-labeled bacteria suspended in antibiotic-free K-SFM were added to the apical compartment of the double-chamber system at an MOI of 10^4^. Bacteria from an overnight culture were labeled with FITC as described previously [19]. To evaluate the effect of the Dual Zinc plus Arginine aqueous solution and dentifrice on the invasive capacity of *P. gingivalis*, the keratinocyte layer was co-incubated with them and the bacteria. The translocation of FITC-labeled bacteria through the keratinocyte barrier was monitored using a Synergy 2 microplate reader by measuring the fluorescence (RFU; excitation wavelength 495 nm; emission wavelength 525 nm) in the medium recovered from the lower chamber following a 24-h incubation in an anaerobic chamber at 37°C. Assays were performed in triplicate in three independent experiments and the means ± SD of a representative set of data is presented.

### Statistical analysis

Statistical analyses were performed using a one-way analysis of variance with a post hoc Bonferroni multiple comparison test (GraphPad Software Inc., La Jolla, CA, USA). All results were considered statistically significant at *p*< 0.05.

## Results

*P. gingivalis* cells caused marked hemolysis of sheep red blood cells in a hemolytic assay (Table 1). Both the Dual Zinc plus Arginine aqueous solution and dentifrice significantly reduced hemolysis. At the lowest dilution tested (1/500), hemolysis was reduced by 47.5% and 37.6%, respectively. The control fluoride dentifrice did not inhibit the hemolysis caused by *P. gingivalis*.

**Table 1.** Effects of the Dual Zinc plus Arginine aqueous solution, the Dual Zinc plus Arginine dentifrice, and the regular fluoride dentifrice on the hemolytic activity of *P. gingivalis*. A value of 100% was assigned to the hemolysis induced by *P. gingivalis* in the absence of compounds. Results are expressed as the means ± SD of triplicate assays from two independent experiments. *, significant inhibition (p < 0.05) compared to control (no compounds).

Compounds	Dilution	Relative hemolysis
None		100%
Dual Zinc plus Arginine aqueous solution	1/500	52.5 ± 1.7%*
	1/1000	57.2 ± 0.6%*
	1/2000	54.3 ± 2.9%*
	1/4000	61.1 ± 0.5%*
	1/8000	87.5 ± 2.2%*
	1/16000	104.6 ± 0.9%
Dual Zinc plus Arginine dentifrice	1/500	62.4 ± 1.2%*
	1/1000	61.1 ± 0.5%*
	1/2000	69.2 ± 0.1%*
	1/4000	74.0 ± 1.2%*
	1/8000	85.6 ± 3.3%*
	1/16000	105.7 ± 1.2%
Control fluoride dentifrice	1/500	114.7 ± 2.3%
	1/1000	94.4 ± 1.9%
	1/2000	106.7 ± 4.3%
	1/4000	106.4 ± 0.8%
	1/8000	98.6 ± 4.9%
	1/16000	92.4 ± 1.6%

**Figure 1.**
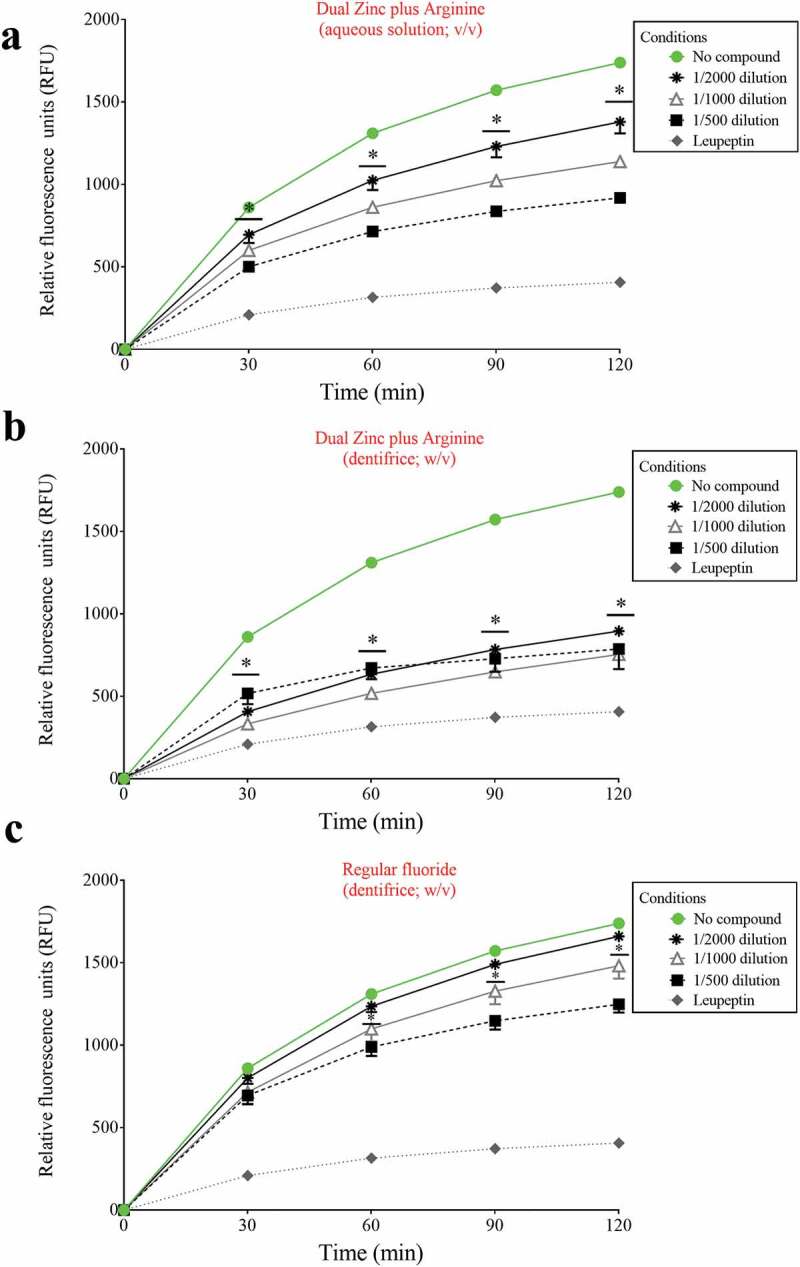
Effects of the Dual Zinc plus Arginine aqueous solution, the Dual Zinc plus Arginine dentifrice, and the regular fluoride dentifrice on collagen degradation by *P. gingivalis*. Results are expressed as the means ± SD of triplicate assays two independent experiments. *, significant decrease (*p* < 0.05) compared to untreated control cells.

We then investigated the ability of the Dual Zinc plus Arginine formulation (aqueous solution and dentifrice) to inhibit the degradation of type I collagen by proteinases present in a culture supernatant of *P. gingivalis*. Significant time- and dose-dependent inhibition was observed with both the Dual Zinc plus Arginine aqueous solution and dentifrice (Figure 1). More specifically, at the lowest dilution tested (1/500) and after a 2-h incubation, the Dual Zinc plus Arginine aqueous solution and the Dual Zinc plus Arginine dentifrice caused a 47.2% and 54.8% inhibition of type I collagen degradation, respectively. The control fluoride dentifrice reduced collagen degradation by 28.2%.

**Figure 2.**
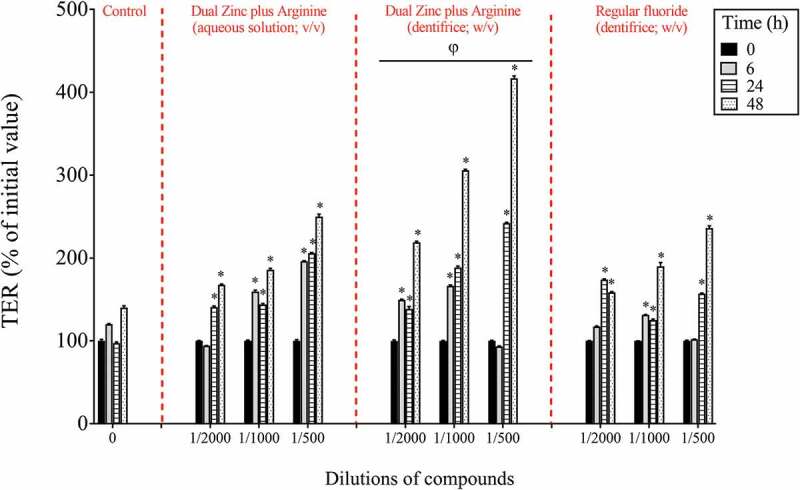
Time- and dose-dependent effects of the Dual Zinc plus Arginine aqueous solution, the Dual Zinc plus Arginine dentifrice, and the regular fluoride dentifrice on gingival keratinocyte tight junction integrity, as determined by monitoring TER. A 100% value was assigned to the TER values at time 0. Results are expressed as the means ± SD of triplicate assays. *, significant increase (*p* < 0.05) compared to untreated control cells. φ, significant increase (*p* < 0.05) compared to the regular fluoride dentifrice.

After investigating the effects of the Dual Zinc plus Arginine formulation (aqueous solution and dentifrice) on *P. gingivalis*, its ability to promote gingival keratinocyte barrier integrity was assessed. Preliminary assays showed that, at the concentrations used, the Dual Zinc plus Arginine aqueous solution and dentifrice did not significantly affect the viability of gingival keratinocytes as determined using a colorimetric MTT assay (data not shown). The ability of the Dual Zinc plus Arginine formulation to modulate the integrity of the gingival keratinocyte tight junction was determined by monitoring TER values over a period of 48 h. As shown in Figure 2, the Dual Zinc plus Arginine aqueous solution and dentifrice induced a significant time-dependent increase in TER. A 24-h treatment of the keratinocytes with the 1/500 and 1/1000 dilutions of the Dual Zinc plus Arginine aqueous solution caused a 2.1- and 1.5-fold increase in TER, respectively, compared to untreated cells. A similar treatment with the Dual Zinc plus Arginine dentifrice caused a 2.5- and 1.9-fold increase in TER, respectively. Under the same conditions, the 1/500 and 1/1000 dilutions of the control fluoride dentifrice caused a 1.6-and 1.3-fold increase in TER, respectively.

**Figure 3.**
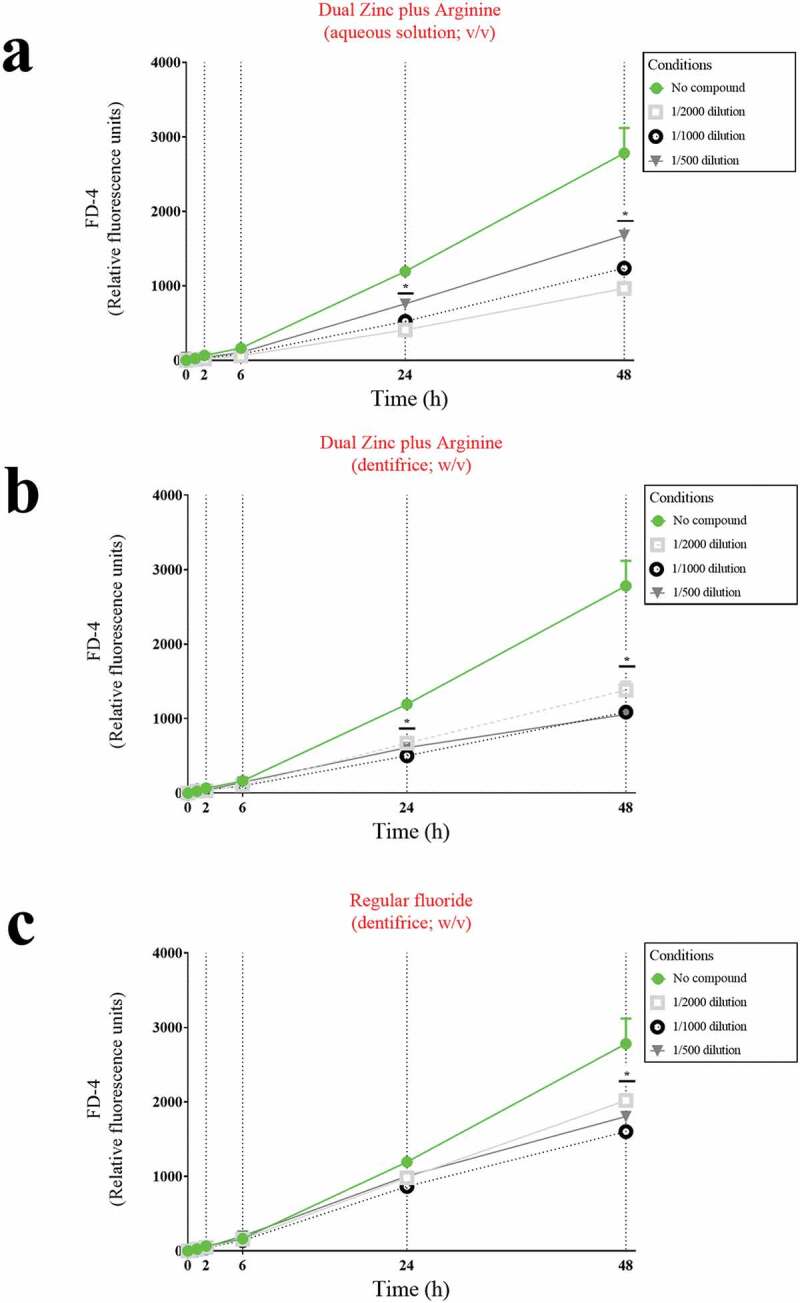
Time- and dose-dependent effects of the Dual Zinc plus Arginine aqueous solution, the Dual Zinc plus Arginine dentifrice, and the regular fluoride dentifrice on the paracellular permeability of gingival keratinocytes to FITC-dextran 4 (FD-4). Results are expressed as the means ± SD of triplicate assays. *, significant decrease (*p* < 0.05) compared to untreated control cells.

To confirm that the Dual Zinc plus Arginine aqueous solution and dentifrice enhanced the function of the keratinocyte barrier, their effect on paracellular permeability was investigated by measuring the apical-to-basolateral transport of FD-4. As shown in Figure 3, the paracellular transport of FD-4 time-dependently increased in the control assay (no compounds). However, in the presence of the the Dual Zinc plus Arginine aqueous solution or dentifrice, the increase in FD-4 transport through the gingival keratinocyte barrier was significantly attenuated. More specifically, following a 24-h treatment, the aqueous solution and dentifrice at the lowest dilution tested (1/500) reduced FD-4 transport by 36.4% and 49.0%, respectively, while the control fluoride dentifrice only reduced FD-4 transport through the barrier model by 15.8%.

**Figure 4.**
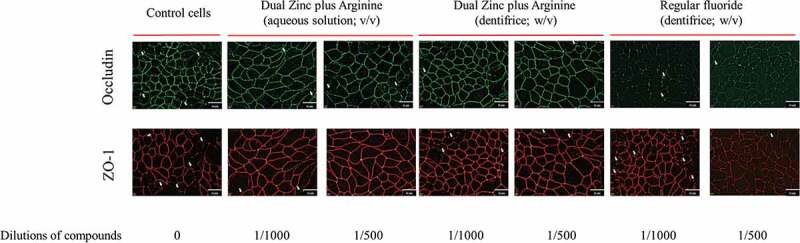
Immunofluorescence staining of the tight junction proteins occludin and zonula occludens-1 of gingival keratinocytes treated for 48 h with the Dual Zinc plus Arginine aqueous solution, the Dual Zinc plus Arginine dentifrice, and the regular fluoride dentifrice. Arrows indicate discontinuities in protein labeling. This analysis was performed three times; a representative experiment and a representative field of this experiment is presented.

We then examined the effect of the 1/500 and 1/1000 dilutions of the Dual Zinc plus Arginine aqueous solution and dentifrice on the distribution of two junction proteins (ZO-1 and occludin) by immunofluorescence. Both the aqueous solution and dentifrice increased the immunolabeling of ZO-1 and occludin in the areas of cell-cell contact (Figure 4), while the regular fluoride dentifrice had few or no effect on the immunolabeling of ZO-1 and occludin.

**Figure 5.**
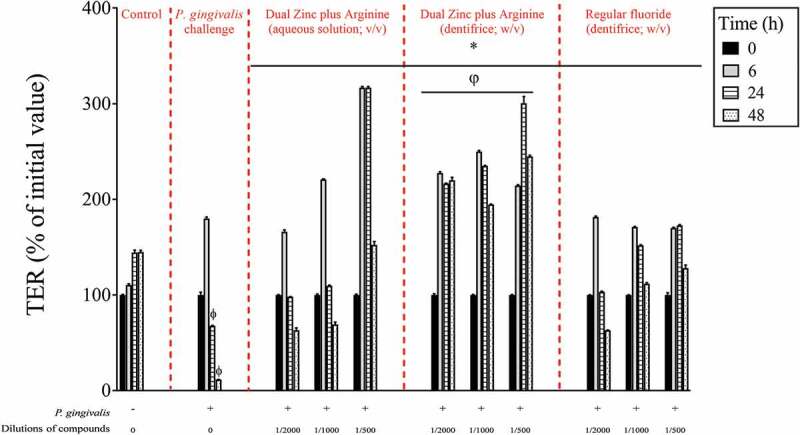
Time- and dose-dependent protective effects of the Dual Zinc plus Arginine aqueous solution, the Dual Zinc plus Arginine dentifrice, and the regular fluoride dentifrice against *P. gingivalis-*mediated damage of gingival keratinocyte tight junction integrity as determined by monitoring TER values. A 100% value was assigned to the TER values at time 0. Results are expressed as the means ± SD of triplicate assays. *, significant increase (*p* < 0.001) compared to *P. gingivalis*-infected cells not treated with compounds. Φ, significant decrease (*p* < 0.05) compared to non-stimulated control cells. φ, significant increase (*p* < 0.05) compared to the regular fluoride dentifrice.

**Figure 6.**
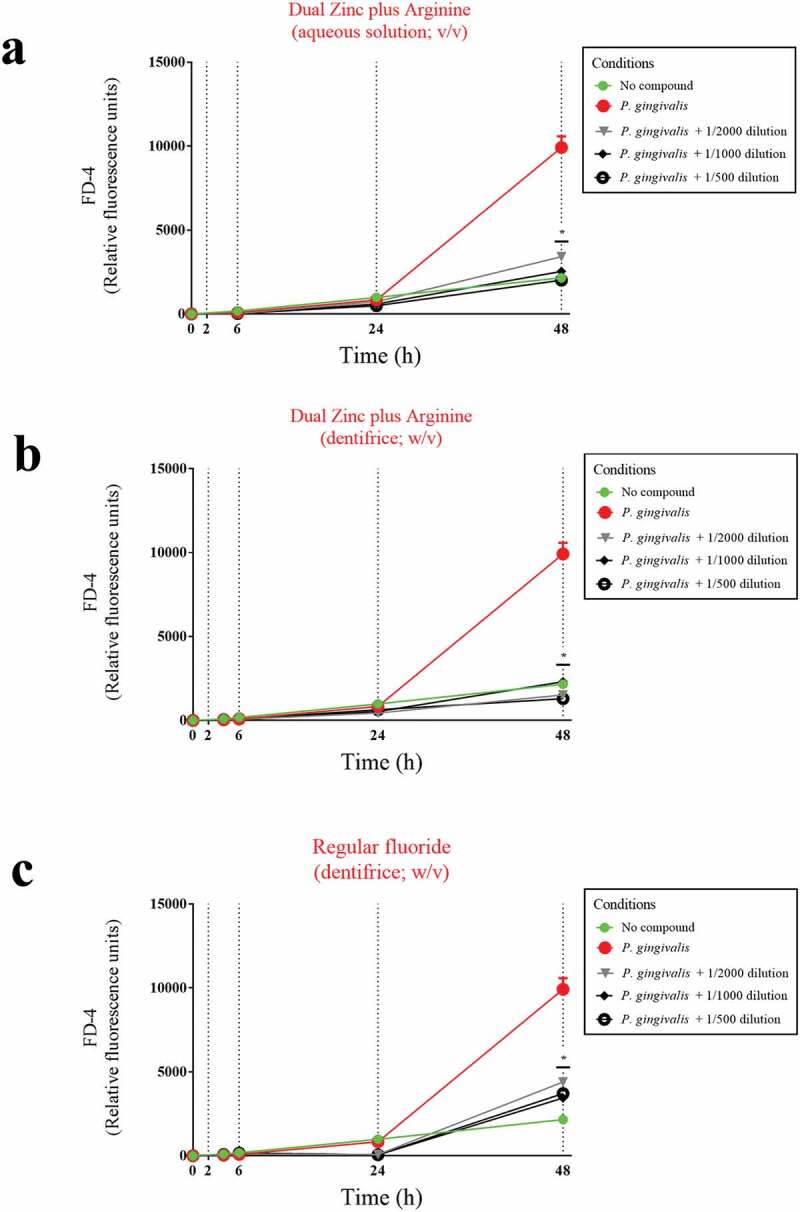
Protective effects of the Dual Zinc plus Arginine aqueous solution, the Dual Zinc plus Arginine dentifrice, and the regular fluoride dentifrice on the paracellular permeability of gingival keratinocytes to FITC-dextran 4 (FD-4) compromised by *P. gingivalis*. Results are expressed as the means ± SD of triplicate assays. *, significant decrease (*p* < 0.05) compared to *P. gingivalis*-stimulated cells.

Since *P. gingivalis* may have a deleterious effect on keratinocyte barrier integrity, we investigated whether the Dual Zinc plus Arginine aqueous solution and dentifrice protect gingival keratinocytes from damage. Treating the keratinocytes with *P. gingivalis* at a MOI of 10^4^ significantly decreased TER. After 24- and 48-h incubations, *P. gingivalis* decreased TER by 53.2% and 92.1%, respectively (Figure 5). Despite the effect of *P. gingivalis* on barrier integrity, it should be noted that no significant loss of cell viability was observed using a MTT assay that determines cell metabolic activity (data not shown). We then examined the protective effect of the Dual Zinc plus Arginine aqueous solution and dentifrice on TER when the keratinocytes were challenged with *P. gingivalis*. As shown in Figure 6, both compounds attenuated the *P. gingivalis*-mediated loss of keratinocyte barrier integrity. More specifically, following a 48-h incubation, a 1/500 dilution of the Dual Zinc plus Arginine aqueous solution and dentifrice reduced the ability of *P. gingivalis* to decrease TER 13.4-fold and 21.4-fold, respectively. A 1/500 dilution of the regular fluoride dentifrice also provided a protective effect, reducing *P. gingivalis*-induced damage by 11.2-fold.

To confirm this protective effect, we investigated the effect of the Dual Zinc plus Arginine aqueous solution and dentifrice on *P. gingivalis*-induced paracellular flux of FD-4 through the keratinocyte barrier (Figure 6). A 48-h treatment with a 1/500 dilution of the Dual Zinc plus Arginine aqueous solution and dentifrice caused a 4.9-fold and 7.6-fold decrease in FD-4 transport, respectively. The regular fluoride dentifrice caused a 2.7-fold decrease in FD-4 transport.

**Figure 7.**
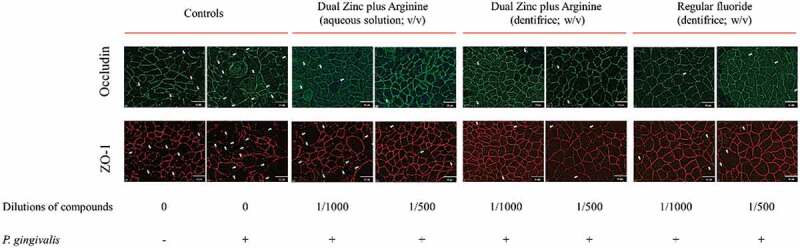
Immunofluorescence staining of the tight junction proteins occludin and zonula occludens-1 in gingival keratinocytes following a 48-h treatment with *P. gingivalis* (MOI = 10^4^) in the absence and presence of the Dual Zinc plus Arginine aqueous solution, the Dual Zinc plus Arginine dentifrice, or the regular fluoride dentifrice. Arrows indicate discontinuities in protein labeling. This analysis was performed three times; a representative experiment and a representative field of this experiment is presented.

ZO-1 and occludin immunostaining was performed to determine whether *P. gingivalis* affects the keratinocyte barrier through the disruption of these two tight junction proteins. As shown in Figure 7, a 48-h treatment of the keratinocytes with *P. gingivalis* (MOI of 10^4^) was associated with a marked decrease in ZO-1 and occludin immunolabeling. ZO-1 and occludin immunolabeling appeared to be less intense and more discontinuous in the cell-cell contacts after a treatment with *P. gingivalis* compared to control cells. However, both the Dual Zinc plus Arginine aqueous solution and dentifrice prevented the discontinuous and less intense immunolabeling of ZO-1 and occludin.

**Figure 8.**
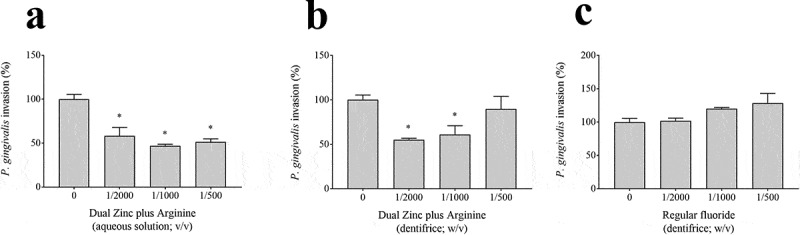
Effects of the Dual Zinc plus Arginine aqueous solution, the Dual Zinc plus Arginine dentifrice, and the regular fluoride dentifrice on the invasion of a gingival keratinocyte barrier by *P. gingivalis*. Results are expressed as the means ± SD of triplicate assays. *, significant decrease (*p* < 0.001) compared to *P. gingivalis*-infected cells not treated with the compounds.

We then determined the effect of the Dual Zinc plus Arginine aqueous solution and dentifrice on the translocation of *P. gingivalis* through the gingival keratinocyte barrier model. The FITC-labeled *P. gingivalis* cells crossed the keratinocyte barrier in a double-chamber system (Figure 8). A 1/1000 dilution of the Dual Zinc plus Arginine aqueous solution and dentifrice significantly reduced the migration of the FITC-labeled *P. gingivalis* cells through the barrier by 53.0% and 39.1%, respectively. The regular fluoride dentifrice caused no significant decrease in the migration of FITC-labeled *P. gingivalis* cells.

## Discussion

Two strategies can be used to promote periodontal health: (1) attenuate/neutralize the pathogenicity of periodontal pathogens, and (2) improve innate immunity by reinforcing epithelial barrier function. In the present study, we investigated the effects of a Dual Zinc plus Arginine aqueous solution and dentifrice on the pathogenic properties of *P. gingivalis* as well as on the barrier function of an *in vitro* gingival epithelium model.

We first showed that the Dual Zinc plus Arginine formulation reduced the hemolytic activity of *P. gingivalis*. The ability of *P. gingivalis* to lyse erythrocytes and release hemoglobin is considered a virulence determinant since it provides an iron source to *P. gingivalis* and other periodontal pathogens and thus promotes their proliferation in subgingival sites. Moreover, hemoglobin has been reported to synergize with *P. gingivalis* lipopolysaccharides to amplify the inflammatory response of human macrophages [20]. As such, the partial inhibition of hemolysis by the Dual Zinc plus Arginine formulation may contribute to reducing the levels of pro-inflammatory mediators in periodontal sites in addition to attenuate proliferation of *P. gingivalis*.

Type I collagen makes up approximately 60% of the tissue volume of periodontal tissues. The collagenolytic activity of *P. gingivalis* has been attributed to the action of its gingipains, which are both secreted and cell-bound [21,22]. We showed that the Dual Zinc plus Arginine formulation dose-dependently reduces collagen degradation by *P. gingivalis*, suggesting that it may contribute to attenuate the tissue destructive process mediated by this periodontal pathogen. Interestingly, previous studies aimed at identifying effective inhibitors of *P. gingivalis* gingipains have shown that both L-arginine [23] and zinc [24], both of which are found in the Dual Zinc plus Arginine formulation, are highly effective in this regard.

The Dual Zinc plus Arginine formulation was found to attenuate two important virulence factors (hemolysin and gingipains) of *P. gingivalis*. Future studies aimed to investigate the antibacterial and anti-biofilm properties of the formulation against *P. gingivalis* should be carried out. Such studies are supported by the fact that Manus et al. [25] recently reported that the Dual Zinc plus Arginine formulation was able to reduce bacterial viability in saliva-derived biofilm models. Evidence was brought that the antibacterial activity resulted from the action of zinc while the presence of arginine disrupts the architecture/porosity of biofilms and allows zinc to penetrate deeper into the biofilm.

The first line of host defense against both opportunistic and pathogenic microorganisms colonizing the oral cavity is the oral epithelium [8,9]. The physical epithelial barrier is composed of closely opposed cells that connect neighboring cells to each other by specialized intercellular tight junctions [26]. These tight junctions seal the paracellular space, blocking the pathway to bacteria and toxins while allowing the flux of water and nutrients. Given the crucial protective role played by the oral epithelial barrier, compounds endowed with a capacity to enhance or protect tissue barrier function are of great interest as potential oral care products. In this regard, we previously reported that plant polyphenols, including green tea catechins [18], black tea theaflavins [27], and blueberry proanthocyanidins [28] improve tight junction integrity in an *in vitro* gingival epithelium model. Although different mechanistic pathways are likely involved, in the present study, we showed that the Dual Zinc plus Arginine aqueous solution and dentifrice significantly enhance the barrier function of a gingival keratinocyte model, as determined by a time-dependent increase in transepithelial electrical resistance and decrease in paracellular permeability. Moreover, the Dual Zinc plus Arginine formulation also increased the immunolabeling of ZO-1 and occludin. The ability of the Dual Zinc plus Arginine formulation to promote gingival keratinocyte barrier function may be associated with the presence of zinc. This is supported by the results of Rybakovsky et al. [29], who recently investigated the effects of various micronutrients on transepithelial electrical resistance in an *in vitro* oral mucosa model and showed that zinc can improve epithelial barrier function. Moreover, in preliminary assays using zinc oxide and zinc citrate, we observed a significant increase in TER in our keratinocyte model although the effects were much less important than with the Dual Zinc plus Arginine formulation (data not shown).

In addition to being a physical barrier against the invasion of the underlying connective tissue by periodontopathogenic bacteria, keratinocytes provide an immunological barrier by secreting antimicrobial β-defensin peptides active against Gram-positive and Gram-negative bacteria [30]. Given that several compounds, including green tea catechins, can increase the innate immunity of oral keratinocytes by inducing human β-defensin secretion [31], it may be of interest to investigate whether the Dual Zinc plus Arginine formulation can increase β-defensin secretion in oral keratinocytes. This would further support the positive impact of the formulation for improving the protective barrier function of the oral epithelium.

*P. gingivalis* has developed various strategies to invade the gingival epithelium and overcome its protective functions [32,33]. In the present study, we showed that *P. gingivalis* can compromise epithelial barrier function by inducing the disorganization of cell-cell interactions as shown by the decrease in TER and increase in FD-4 transport. *P. gingivalis* also affected the distribution of two major tight junction proteins (zonula occludens-1 and occludin). This is in agreement with the results of Katz et al. [34], who used Western blotting to show that *P. gingivalis* (cells and supernatant) can cleave purified occludin. These effects may allow bacteria to reach and damage the underlying connective tissue. The Dual Zinc plus Arginine formulation protected the gingival keratinocyte barrier against *P. gingivalis*-mediated damage. This protective effect may rely on the ability of the zinc to enhance the gingival epithelium barrier function observed in the present study. It may also, at least in part, result from the ability of the Dual Zinc plus Arginine formulation to inhibit *P. gingivalis* gingipain activity. Groeger et al. [11] provided evidence that these proteolytic enzymes are involved in the degradation of cell-to-cell junctions and the disruption of the epithelial barrier. In a recent study, Sarkar et al. [35] used *in vivo* (mouse) and *in vitro* models to demonstrate that zinc can protect the intestinal epithelial barrier from damage induced by the pathogen *Shigella flexneri*. This protective effect was associated with a redistribution of two tight junction proteins (claudin−2 and −4) to the plasma membrane.

The intercellular spaces of the stratified oral epithelium offer a pathway for *P. gingivalis* to invade tissues during periodontitis [36,37]. We thus investigated the effect of the Dual Zinc plus Arginine formulation on the translocation of *P. gingivalis* through an *in vitro* model of the gingival epithelium. We used FITC-labeled bacteria to show that the formulation reduced the migration of *P. gingivalis* through the gingival keratinocyte barrier in a double-chamber system. Since gingipains contribute to the invasive capacity of *P. gingivalis*, their inhibition by the formulation may be responsible, at least in part, in a reduced translocation through our *in vitro* model of the gingival epithelium.

In conclusion, the present study provided clear *in* *vitro* evidence that the Dual Zinc plus Arginine formulation, in an aqueous solution or in a dentifrice, may offer benefits for periodontal health through its ability to attenuate the pathogenic properties of *P. gingivalis* and enhance epithelial barrier function. Among the limitations of the study was the fact that only one keratinocyte cell line and one *P. gingivalis* strain were used. Moreover, clinical trials are necessary to determine whether the beneficial effects observed with Dual Zinc plus Arginine formulation in our *in* *vitro* models can be achieved in an *in vivo* situation.
