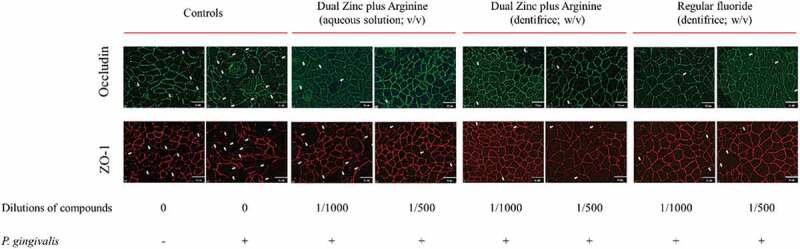# Correction

**DOI:** 10.1080/20002297.2020.1813916

**Published:** 2020-09-02

**Article title**: A Dual Zinc plus Arginine formulation attenuates the pathogenic properties of *Porphyromonas gingivalis* and protects gingival keratinocyte barrier function in an *in vitro* model

**Authors**: Amel Ben Lagha, Ying Yang, Harsh M. Trivedi, James G. Masters and Daniel Grenier

**Journal**: *Journal of Oral Microbiology*

**DOI**: 10.1080/20002297.2020.1798044

We have been notified by the authors that Figure 7 had been incorrectly updated in the published article. Figure 7 has now been corrected online as shown below. This correction has not changed the description, interpretation, or the original conclusions of the article.

**Figure:**